# DOK1 and DOK2 regulate CD8 T cell signaling and memory formation without affecting tumor cell killing

**DOI:** 10.1038/s41598-024-66075-0

**Published:** 2024-07-01

**Authors:** Vladimir Laletin, Pierre-Louis Bernard, Camille Montersino, Yuji Yamanashi, Daniel Olive, Rémy Castellano, Geoffrey Guittard, Jacques A. Nunès

**Affiliations:** 1 Centre de Recherche en Cancérologie de Marseille, CRCM, Immunity and Cancer Team, Institut Paoli-Calmettes, Inserm, CNRS, Aix Marseille University, Marseille, France; 2 Centre de Recherche en Cancérologie de Marseille, CRCM, TrGET Pre-Clinical Assay Platform, Institut Paoli-Calmettes, Inserm, CNRS, Aix Marseille University, Marseille, France; 3 Division of Genetics, The Institute of Medical Science, The University of Tokyo, 4-6-1 Shirokanedai, Minato-ku, Tokyo, 108-8639 Japan

**Keywords:** Signal transduction, Tumour immunology, CD8-positive T cells

## Abstract

Targeting intracellular inhibiting proteins has been revealed to be a promising strategy to improve CD8^+^ T cell anti-tumor efficacy. Here, we are focusing on intracellular inhibiting proteins specific to TCR signaling: DOK1 and DOK2 expressed in T cells. We hypothesized that depletion of intracellular inhibition checkpoint DOK1 and DOK2 could improve CD8^+^ T-cell based cancer therapies. To evaluate the role of DOK1 and DOK2 depletion in physiology and effector function of CD8^+^ T lymphocytes and in cancer progression, we established a transgenic T cell receptor mouse model specific to melanoma antigen hgp100 (pmel-1 TCR Tg) in WT and *Dok1*/*Dok2* DKO (double KO) mice. We showed that both DOK1 and DOK2 depletion in CD8^+^ T cells after an in vitro pre-stimulation induced a higher percentage of effector memory T cells as well as an up regulation of TCR signaling cascade- induced by CD3 mAbs, including the increased levels of pAKT and pERK, two major phosphoproteins involved in T cell functions. Interestingly, this improved TCR signaling was not observed in naïve CD8^+^ T cells. Despite this enhanced TCR signaling essentially shown upon stimulation via CD3 mAbs, pre-stimulated *Dok1*/*Dok2* DKO CD8^+^ T cells did not show any increase in their activation or cytotoxic capacities against melanoma cell line expressing hgp100 in vitro. Altogether we demonstrate here a novel aspect of the negative regulation by DOK1 and DOK2 proteins in CD8^+^ T cells. Indeed, our results allow us to conclude that DOK1 and DOK2 have an inhibitory role following long term T cell stimulations.

## Introduction

CD8^+^ T cells have a key role in tumor eradication through their capacity to specifically recognize tumor antigens and to secrete potent effector molecules for tumor cell killing such as IFNγ, TNFα and granzymes^1,2^. The potential of these cells is a foundation for modern approaches of cancer immunotherapy. Several CD8^+^ T cell-based methods were proposed to fight cancers: CAR-T cells, tumor infiltrating lymphocytes (TILs) and vaccine-based approaches^3^. Despite the potency of these methods the majority of patients do not respond. The reason underlying this phenomenon is associated with the negative regulatory tumor microenvironment, inhibitory ligands and diminished T cell antigen receptor (TCR) signaling^4–8^. Therefore, new methods to improve CD8^+^ T cell-based immunotherapies of cancer are required. One promising approach is to improve TCR signaling targeting intracellular inhibiting proteins^9,10^.

TCR signaling is crucial for T cell activation, differentiation and cytotoxicity. It is initiated by cell-surface expressed TCR followed by a complex step of intracellular signal transduction and potentiation that implicates numerous proteins^11^. Protein tyrosine kinases, such as Lck, Fyn and ZAP-70 are involved in proximal to TCR signal transduction, whereas adaptor proteins LAT, Grb2 and SLP-76 and intracellular signal transducers PI3K and RAS form a key signalosome for distal to TCR signal potentiation^12,13^. Finally, these TCR encoding signals lead to activation of two main effector pathways in T cell activation, RAS/ ERK-1/2 and PI3K/ AKT signaling pathways that could be easily detected by the ERK-1/2 and AKT phosphorylation status. Upon TCR engagement and activation, naïve T cells undergo a maturation process that give rise to memory T cells. This maturation is associated to changes in TCR signal encoding, activation and expression of surface markers^14,15^. Therefore, TCR signaling pathway may differ between naïve and memory, CD4^+^ and CD8^+^ T cells^16–20^.

Previously it was shown that targeting an intracellular TCR-signaling inhibiting protein CISH could improve TCR activation and tumor clearing in tumor bearing mice model^21,22^. Furthermore, clinical studies invalidating CISH in CD8^+^ TILs via CRISPR/Cas9 approach prior to adoptive cell transfer are ongoing. Thus, a new concept of cancer immunotherapies by targeting intracellular inhibiting proteins is emerging. Here we investigated the potential of intracellular TCR-signaling inhibiting proteins DOK1 and DOK2 to improve CD8^+^ T cell activation and cytotoxicity against tumors.

Discovered as tumor suppressor gene products, DOK1 and DOK2 adaptor proteins are two members of DOK family proteins that are constitutively expressed in T cells^23–27^.DOK1 and DOK2 expression levels increase upon T cell maturation^28^. TCR engagement induces the phosphorylation of DOK1 and DOK2^28–30^. DOK1 and DOK2 proteins are implicated in negative regulation of TCR signaling as their deficiency improves TCR-mediated cytokine production and proliferation in T-cell lines and in mouse CD4^+^ T lymphocytes^28,29^. Upon DOK1 and DOK2 tyrosine phosphorylation, some DOK-interacting proteins such as RasGAP, SHIP and Csk proteins are involved in negative feedback loops for TCR signal transduction^12,23,26–29,31,32^. Upon TCR engagement, the loss of DOK1 and DOK2 in CD4^+^ T cells increases both early phosphorylation events such as ZAP-70 and LAT and distal phosphorylation events as ERK-1/2 and AKT^28,29^. For the CD8^+^ T cell compartment. Previously, it has been reported that the CD3 ligation induces the DOK2 tyrosine phosphorylation in a human cytotoxic T cell clone^29^. The analysis of CD8^+^ T cells in in vivo viral infection mouse model showed higher production of IFNγ, TNFα and granzyme B in *Dok1*/*Dok2* double knockout (DKO) mice, without affecting TCR signaling^30^.

To further understand how DOK1 and DOK2 regulate CD8^+^ T cell activity and especially their cytotoxic function against cancer cells, we crossed *Dok1*/*Dok2* DKO mice with pmel-1 TCR transgenic mice^22^. We then investigated the role of DOK1 and DOK2 in physiology, TCR signaling and activation of naïve and primed CD8^+^ T cells and cytotoxic capacity against tumor cells. Primed *Dok1*/*Dok2* DKO CD8^+^ T cells showed increased TCR signaling evaluated by ERK-1/2 and AKT phosphorylation that was not observed in naïve CD8^+^ T cells and acquired effector memory phenotype upon CD8^+^ T cell amplification. However, we detected no difference in activation, cytokine production or cytotoxicity against tumor cells.

We demonstrate, here, a novel aspect of negative regulation by DOK1 and DOK2 proteins in CD8^+^ T cells. Indeed, our results allow us to conclude that DOK1 and DOK2 have a slight inhibitory role following longer term stimulations. However, these adaptor proteins do not appear to be major candidates for the development of in vitro enhanced T cell strategies for cancer immunotherapy.

## Materials and methods

### Mice and cell lines

#### Mice

Generation of *Dok1*/*Dok2* DKO mice was previously reported^33^. Pmel-1 TCR transgenic mice, were a kind gift from Nicholas P. Restifo (NCI, Bethesda, USA)^34^. Mice were crossed to generate *Dok1*/*Dok2* DKO Pmel-1 transgenic mouse strain. C57BL/6 Ly5.1 were purchased from Janvier Labs and housed in our animal facility at least one week before starting the experimental protocol. All mice were crossed, housed and genotyped according to the guidelines of Committee for Animal Experimentation of Marseille and in accordance with European Directive 2010/63/EU. The experimental protocol was approved by an Institutional Animal Care and Use Committee (the Structure du bien-être animal (SBEA) du Centre de Recherche en Cancérologie de Marseille (CRCM) and the Comité d’Ethique en Expérimentation Animale n°14 – CEEA 14). Male and female mice were used between the ages of 6–12 weeks. Mice were bred and maintained under specific pathogen-free conditions at the Centre de Recherche en Cancérologie de Marseille (CRCM) animal facility.

#### Genotyping

Pmel-1 TCR genotyping was performed as described previously^22^ using following primers: 5Chr2pmel: 5′ CTT TAG ACC TCC GGC ACT GTT GC 3′; 3Chr2pmel: 5′ GCA AGT AGC AGT GTA TCA AAT ATG C 3′; 3PmelTCRb: 5′ GTA GCT TTG TAA GGC TGT GGA GAG 3′, with expected bands band sizes at 280 and 300 bp. Dok1 and Dok2 genotyping was performed using following primers: Dok1_F bisGAAATGACATCTTTCAGGCAGTTGAGGC; Dok1_R bis GAGTCTGTCAGCTTGGTTTTCAGTAACT; Dok2_F GTTCGCAGCCGTGTTATATGGAGAGTCT; Dok2_R GAAAGCCAACAGGCAGATGGCCTGTAT, with expected bands on 351 and 261 bp for Dok1 and Dok2 respectively.

#### Cell culture

B16 melanoma (H-2D^b^), a mouse melanoma, transduced retrovirally to express human glycoprotein 100 (hgp100) with human residues at position 25–27 was a kind gift from the team of Nicholas P. Restifo (NCI, Bethesda, USA)^35^. WT B16 melanoma cells and B16 expressing hgp100 were grown in DMEM (Gibco) 20% FCS, 1% NEAA (Gibco), and 1% Sodium butyrate (Sigma-Aldrich).

### In vitro CD8^+^ T cell expansion

Naïve CD8^+^ T lymphocytes were isolated from splenocytes by magnetic bead negative selection per the manufacturer’s protocol (Invitrogen). In accordance with the technical manual, the percentage of CD8 + T cells upon selection was around 80%. CD8^+^ T cell expansion was performed in Gibco™ RPMI 1640 medium 10%FCS, 50 μM β-mercaptoethanol (Sigma-Aldrich). Splenocytes were cultured in presence of hgp100_25–33_ peptide, KVPRNQDWL (AnaSpec, CliniSciences) at 100 ng/mL and human IL-2 (100 IU/mL, Roche) or expanded by plate-bound CD3ε mAb at 2 μg/mL (clone 145-2C11, BD Pharmingen) and soluble CD28 mAb at 1 μg/mL (clone 37.51, BD Pharmingen) and human IL-2 (100 IU/ml) for 3 days. This was followed by 2 days of human IL-2 (100 IU/ml) maintenance.

### Flow cytometry

For cell phenotyping a single cell suspension was prepared. Red blood cells lysis was performed if necessary, using 1X ACK lysis buffer (Gibco). Extracellular staining was performed for 30 min at 4 ℃. When necessary, intracellular staining was performed by use of the FoxP3/Transcription Factor Staining Buffer Set (eBioscience) according to the manufacturer’s instructions. Used antibodies for CD8^+^ T cell phenotyping: CD4-PerCP-Cy5.5 (BioLegend, #100539), CD3-PE-Cy7 (eBioscience, #25-0031-82), CD8-APC-EF780 (Invitrogen #47-0081-82), CD62L-APC (Invitrogen, #17-0621-81), CD44-FITC (BD Pharmingen, #561859), CD62L-eF450 (Invitrogen, #48-0621-82), CD44-AF700 (Invitrogen #56-0441-82), CD69-PE (BD Pharmingen, # 553237). Gating strategies for effective flow cytometry data analysis are described (Fig. S1A). For phosphoflow experiments cells were immediately fixed by FoxP3/Transcription Factor Staining Buffer Set (eBioscience) for 10 min at 37 ℃ after stimulation and labeled by pErk-AF488 (Cell Signaling, #13214) and pS6-APC (Cell Signaling, #14733) antibodies. Dead cell exclusion was done by LIVE/DEAD™ Fixable Aqua Stain (Thermo Fisher Scientific, L34957). All data were acquired on LSRII, Fortessa, (BD Biosciences) and analyzed with FlowJo software (Tree Star, Ashland, OR, USA).

### CD8^+^ T cell cross linking stimulation

For TCR stimulation naïve and primed CD8^+^ T cells were incubated for 20 min at 4 ℃ with biotinylated CD3ε mAb (5 and 1 μg/mL, BD Biosciences #553060). Cells were washed and stimulated for the indicated time by adding streptavidin (20 μg/mL, final concentration) at 37 ℃. For peptide stimulations purified primed CD8^+^ T cells were stimulated by hgp100_25–33_ peptide, KVPRNQDWL (AnaSpec, CliniSciences) at 1000 ng/mL.

### Western blotting

Stimulated cells were lysed at 4 ℃ for 10 min in 1% NP-40 lysis buffer (50 mM Tris pH 7.4, 150 mM NaCl, 5 mM EDTA, protease inhibitor cocktail (Roche #11836170001), 1 mM Na_3_VO_4_, 0.1% SDS). Samples were resolved by 10% SDS–polyacrylamide gel electrophoresis experiments. Blots were incubated overnight at 4 ℃ with the corresponding primary antibody directed p-AKT (Cell Signaling Technology #9271), Akt (Cell Signaling Technology #9272), p-Erk1/2 (Cell Signaling Technology #4377), Erk-1/2 (Cell Signaling Technology #9102) and β-Actin (Cell Signaling Technology #3700). Blots were incubated with corresponding peroxidase–conjugated secondary antibodies (Millipore #DC02L; #DC03L) for 1 h at room temperature. ECL (enhanced chemiluminescence; SuperSignal West Pico and SuperSignal West Femto, Pierce) was used to visualize protein bands. Full-length gels and blots are shown in a supplementary information file.

### Cytokine production

Primed mouse CD8^+^ T cells were cultured alone, or with B16 WT or B16-hgp100 target cells (1:10 E:T ratio). Following the supplier recommendations, after four hours of incubation at 37 ℃ in the presence of FITC-conjugated CD107a antibody (BD Pharmingen #553793), GolgiStop™ and GolgiPlug™ (BD Biosciences), cells were stained and the percentages of CD8^+^ T cells positive for CD107a, TNF-α (APC, Invitrogen, #17-7321-82) and IFN-γ (PE, Invitrogen, #12-7311-81) were measured by flow cytometry.

### Cytotoxicity

Target B16 cells were stained with 4 μM of Cell Proliferation Dye eFluor™ 670 (Life Technologies) according to manufacturer's instructions. Primed CD8^+^ T cells were then incubated with target cells for four hours at 37 ℃ at different effector to target (E:T) ratios. Target cell killing was measured using CellEvent™ Caspase-3/7 Green Detection Reagent (Life Technologies) and analyzed by flow cytometry.

### Conjugate formation

This method has been adapted from a previous report on NK cells^36^. Here, primed CD8^+^ T cells were incubated for 30 min on ice with CD8-APC-EF780 antibody (Invitrogen, #47-0081-82) in serum-free RPMI medium. They were then washed and resuspended at 20.10^6^ cells per ml. 100 μL of cell suspension was then added to 100 μL of labeled with Cell Trace Violet (V450) (Invitrogen) B16 WT or B16-hgp100 cells (at 20.10^6^ cells per mL) and centrifuged at 1,500 rpm (4 ℃). After removing 150 μL of supernatant, cells were stimulated by incubation at 37 ℃ for 0, 5, or 10 min. Reactions were stopped by adding ice-cold phosphate-buffered saline. Conjugates were detected by flow cytometry as double positive CD8 + V450 + events.

### In vivo migration assays

Primed CD8^+^ T cells isolated from WT or *Dok1*/*Dok2* DKO mice were loaded with Cell Trace Violet Stain (Life Technologies #C34557) or Cell Trace Far Red DDAO (Life Technologies #C34553). As described previously^37^, cells were mixed at a ratio of 1:1 and 10.10^6^ cells were *i.v.* injected in C57BL/6 mice. Then, 1 h later, recipient mice were euthanized, and blood, spleen, and (inguinal, axillary and brachial) lymph nodes were removed for quantification of Cell Trace Violet-labeled and Cell Trace Far Red-labeled T cells by flow cytometry.

### Adoptive cell transfer

For immunotherapy, C57BL/6 Ly5.1 (Janvier Labs) were implanted with subcutaneous B16 melanoma (5.10^5^cells). At 10 days after tumor implantation, mice (n = 9 for “PBS” condition and n = 8 for the two adoptive cell transfer conditions) were sub-lethally irradiated (600 cGy), randomized, and injected intravenously with 5.10^5^ Pmel-1 *Dok1*/*Dok2* DKO or WT Ly5.2 primed CD8^+^ T cells and received intraperitoneal injections of human IL-2 (Aldesleukin, Clinigen, NL) in PBS (6.10^4^ IU/0.5 mL) once daily for 3 days starting on the day of T cell transfer. As a control, IL-2 is also injected in the “PBS” conditions. Mice with tumors greater than 400 mm^2^ or in illness state were euthanized (Application for a project authorization of animal experimentation: DAP #28902).

### Statistical analysis

The data were expressed as the mean ± SEM. Prism 5.03 software (GraphPad, San Diego, CA, USA) was used for all statistical analysis. Statistical significance between control (WT) and *Dok1*/*Dok2* DKO groups was determined by two-tailed Student *t*-test. *P < 0.05, **P < 0.01, ***P < 0.001. Mantel Log-rank test was used to compare survival curves between PBS and WT or *Dok1*/*Dok2* DKO adoptive cell transfer (*, p < 0,05).

### Statements

The study is reported in accordance with ARRIVE guidelines ( https://arriveguidelines.org ).

Furthermore, the experiments were conducted in accordance with the Guidelines of the European Union Council (2010/63/EU) and French legislation for the use of laboratory animals (project authorization of animal experimentation: DAP #28902).

## Results

### Primed *Dok1*/*Dok2* DKO CD8^+^ T lymphocytes have an effector/memory phenotype

**Figure 1:**
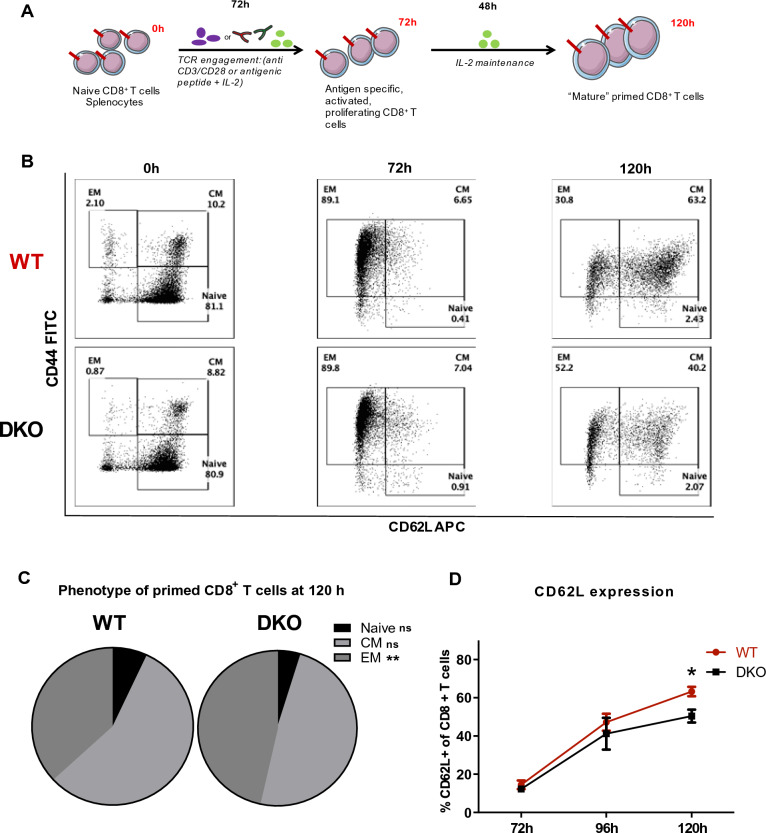
The loss of Dok1 and Dok2 appears to promote an effector/memory phenotype in primed CD8^+^ T cells. (**A**) Used CD8^+^ T cell expansion protocol: 3 days of TCR engagement with anti-CD3 2 µg/ml and CD28 1 μg/ml or hgp100 peptide (100 ng/ml) in the presence of IL-2 100 UI/ml; following by 2 days of maintenance on IL-2 (100UI/ml). Splenocytes were used for expansion in panels B-D. (**B**) Representative scatter plots showing the phenotype of primed CD8^+^ T cells at 0, 72 and 120 h of (CD3 + CD28) expansion (n = 12). (**C**) Effector memory (EM: CD44 + CD62L-), Central memory (CM: CD44 + CD62L +) and Naïve (CD44-CD62L +) proportion in primed CD8^+^ T cells at 120 h of (CD3 + CD28) expansion (n = 15). (**D**) Expression of CD62L during (CD3 + CD28) expansion of CD8 + T cells measured by flow cytometry (n = 3). Error bars, SEM. *, p < 0,05; **, p < 0,01 by Student *t*-test.

To understand how DOK1 and DOK2 regulate CD8^+^ T cell activity and especially their cytotoxic function against cancer cells, we crossed *Dok1*/*Dok2* DKO mice with pmel-1 TCR transgenic mice. We first tested the impact of DOK1 and DOK2 deletion on naïve and in vitro amplified CD8^+^ T cells. *Dok1*/*Dok2* DKO and WT resting CD8^+^ T cells show similar proportions of CD4^+^ and CD8^+^ T cells, naïve (CD62L + CD44-), central memory (CD62L + CD44 +) and effector memory (CD62L-CD44 +) T cell subsets. To prime cells, naïve CD8^+^ T lymphocytes from spleen were purified and then expanded for 5 days, with anti-CD3, anti-CD28 or hgp100 and IL-2 for 3 days followed by 2 days in IL-2 only. Similar protocol using splenocytes is applied for hgp100 peptide treatment (Fig. 1A). T cell subset phenotype was followed over the time at day 3 and day 5 by flow cytometry. Although no difference in proportion of Naïve (CD44-CD62L +), Central memory (CD44 + CD62L +) and Effector/Memory (CD44 + CD62L-) was observed in unstimulated CD8^+^ T cells or at day 3 of expansion, (Fig. 1B) we noticed that *Dok1*/*Dok2* DKO CD8^+^ T cells had a higher proportion of effector memory cells compared to WT cells at the day 5 of expansion (Fig. 1C). We identified the difference of CD62L expression in WT and *Dok1*/*Dok2* DKO CD8^+^ T cells between day 3 and day 5 (Fig. 1D). Therefore, DOK1 and DOK2 regulate the formation of memory CD8^+^ T cells.

### ***Dok1*** and ***Dok2*** invalidation improves TCR signaling in primed CD8^+^ T cells

**Figure 2:**
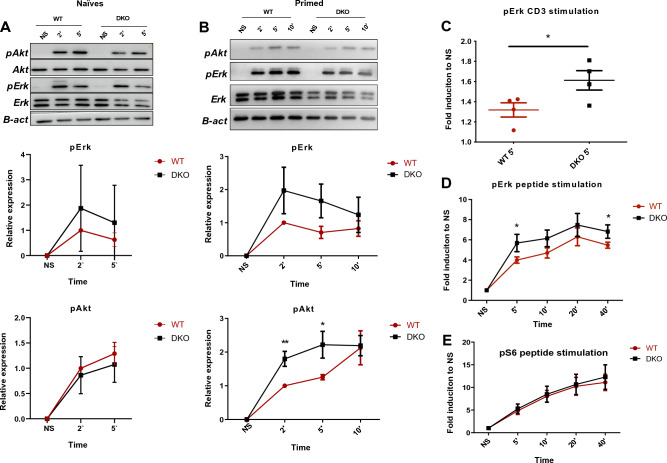
The loss of Dok1 and Dok2 improves TCR signaling in primed CD8 + T cells. Naïve and primed CD8 + T cells were stimulated by anti-CD3 5 µg/ml. (**A**) Representative immunoblots of naïve cells stimulated with anti-CD3 for 2 and 5 min. Full-length blots are shown in a supplementary information file. Normalized quantification of pErk and pAkt induction is shown by using β-actin as the control loading (n = 3). (**B**) Representative immunoblots of primed CD8 + T cells stimulated with anti-CD3 for 2, 5 and 10 min. Normalized quantification of pErk and pAkt induction is shown by using β-actin as the control loading (n = 4). (**C**) pErk induction in primed CD8 + T cells after 5 min of stimulation by anti-CD3 via phospho-flow detection method (n = 4). (**D**) From splenocytes, primed CD8 + T cells were stimulated with hgp100 peptide 1 µg/ml for 5, 10, 20 and 40 min via phospho-flow detection method for pErk or (**E**) for pS6 (n = 5). Error bars, SEM. *, p < 0,05, **, p < 0,01 by Student *t*-test.

We, then, sought to explore the role of DOK1 and DOK2 invalidation in TCR signaling. We used two doses (5 and 1 μg/mL) of biotinylated anti-CD3 to determine the optimal dose for CD8^+^ T cell stimulation (Fig. S1C). *Dok1*/*Dok2* DKO and WT CD8^+^ T cells purified from spleen were stimulated with biotinylated anti-CD3 and cross-linked with streptavidin during the indicated time. After cell lysis the levels of pErk and pAkt were evaluated by Western blot (WB) analysis. Naïve WT and DKO CD8^+^ T cells show the same level of pErk and pAkt upon TCR stimulation (Fig. 2A).

To determine whether the loss of DOK1 and DOK2 affects TCR signaling in primed CD8^+^ T cells, similar experiments were performed. Primed *Dok1*/*Dok2* DKO CD8^+^ lymphocytes showed an upregulation of pErk and pAkt expression compared to WT CD8^+^ T cells upon TCR stimulation, although only pAkt appeared to be statistically significant (Fig. 2B). Subsequently, phosphoflow experiments were performed. Primed CD8^+^ T cells were stimulated with biotinylated anti-CD3 and cross-linked with streptavidin during 5 min. Phosphorylation of ERK1/2 was detected by flow cytometry (Fig. 2C). Again, pErk expression was increased in primed *Dok1*/*Dok2* DKO CD8^+^ T cells compared to WT cells, confirming our WB experiments.

To ensure that this TCR signaling improvement in *Dok1*/*Dok2* DKO primed CD8^+^ T lymphocytes is conserved with a different TCR stimulation setting, we performed a stimulation with hgp100 peptide recognized by the Pmel-1 TCR. Cells were peptide-stimulated, immediately fixed and stained with antibodies against pErk and pS6. Flow cytometry analysis revealed a small increase of pErk induction in *Dok1*/*Dok2* KO primed CD8^+^ T lymphocytes compared to WT CD8^+^ T cells (Fig. 2D). No difference in pS6 expression (Fig. 2E) or in CD69 cell surface expression (Fig. S1B) was found.

Similarly, we performed immunoblot analysis of primed CD8^+^ T cells lysates after a stimulation with hgp100 peptide. We did not notice any difference in pErk and pAkt expression levels between WT and DKO cells (Fig. S2A–C).

Altogether, these findings suggest that DOK1 and DOK2 deficiency enhances TCR signaling especially upon CD3 mAb stimulation. This effect was only observed when CD8^+^ T cells were primed in vitro.

### ***Dok1***/***Dok2*** DKO and WT CD8^+^ T cells show similar cytotoxicity in vitro

**Figure 3:**
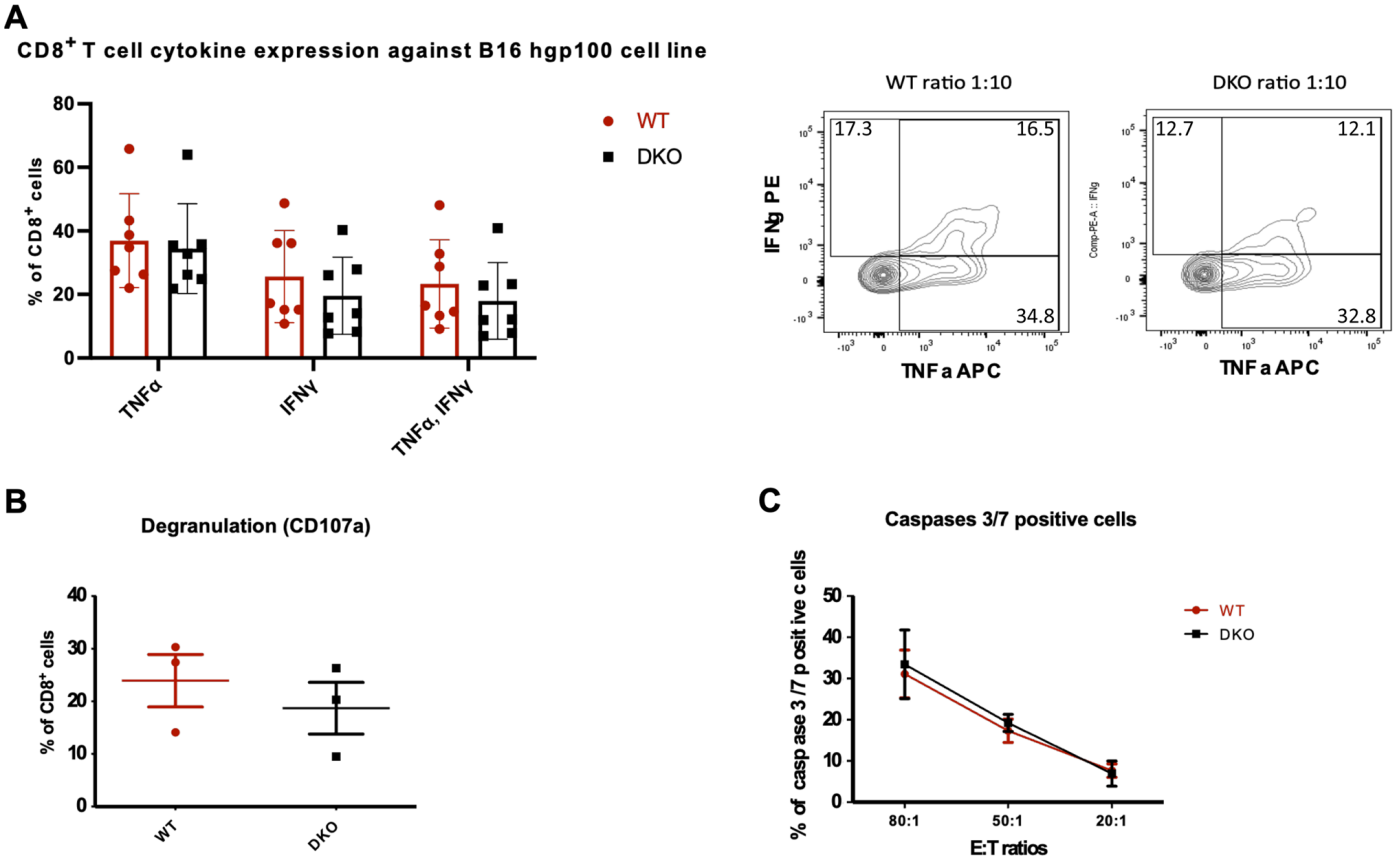
(**A**) The loss of Dok1 and Dok2 does not affect cytotoxic function of primed CD8 + T cells. Cytokine production by primed CD8 + T cells at 1:10 E:T ratio after 4 h of co-culture with B16 hgp100 target cells measured by flow cytometry (n = 7). In the right part of this panel, representative contour plots are shown. (**B**) Degranulation measured by CD107a expression by primed CD8 + T cells at 1:10 E:T ratio after 4 h of co-culture with B16 hgp100 target cells measured by flow cytometry (n = 3). (**C**) Caspases 3/7 activation in B16 hgp100 cells after co-culture with primed CD8 + T cells in different E:T ratios measured by flow cytometry (n = 3).

To assess CD8^+^ T cell cytotoxicity a murine B16 melanoma cell line expressing constitutively hgp100 antigen was used. Primed pmel-1^+^ CD8^+^ T cells (corresponding here to the primed WT CD8^+^ T cells) can recognize hgp-100 antigen at the surface of B16-hgp-100 expressing cells but not when the peptide is not expressed. Primed *Dok1*/*Dok2* DKO and WT CD8^+^ T cells were co-cultured with B16-hgp100 cells at indicated effector/Target (E/T) ratio. Expression of IFN-γ and TNF-α was detected by flow cytometry (Fig. S3A). Surprisingly, *Dok1*/*Dok2* DKO and WT CD8^+^ T cells expressed the same level of IFN-γ and TNF-α for all tested E/T ratios (Fig. 3A and data not shown). Likewise, degranulation marker CD107a showed also similar expression between *Dok1*/*Dok2* DKO and WT CD8^+^ T cells (Fig. 3B).

Next, the capacity of primed CD8^+^ T cells to kill B16-hgp100 cells was analyzed by measuring caspase 3/7 activation in target cells after 4 h of co-culture. Whatever the ratio tested, WT and *Dok1*/*Dok2* DKO primed CD8^+^ T cells displayed a similar cytotoxic activity (Fig. 3C).

Therefore, these data suggest that *Dok1*/*Dok2* DKO and WT CD8^+^ T cells show similar cytotoxicity in vitro.

### Primed ***Dok1/Dok2*** DKO CD8^+^ T cells do not improve survival of tumor-bearing mice

Finally, we evaluated the transfer of primed CD8^+^ T cells to provide a significant benefit survival for tumor-bearing mice. We made a subcutaneous B16-hgp100 tumor injection and 10 days after we performed an adoptive cell transfer of WT and *Dok1*/*Dok2* DKO CD8^+^ T cells. PBS injection was used in a control group. We found that adoptive cell transfer improved a survival of tumor bearing mice, however, there was no difference between WT and *Dok1*/*Dok2* DKO group (Fig. S4). In line with the in vitro experiments, *Dok1* and *Dok2* invalidations do not improve primed CD8^+^ T cell cytotoxicity in the context of adoptive cell transfer.

### ***Dok1***/***Dok2*** DKO and WT CD8^+^ T cells show similar effector: target conjugate formation and migration.

Since, *Dok1*/*Dok2* DKO primed CD8^+^ T lymphocytes have fewer expression of CD62L adhesion molecule (Fig. 1D), we hypothesized that it could impact the formation of effector-target cell conjugates or migration. For migration experiment WT and *Dok1*/*Dok2* DKO primed CD8^+^ T cells were labeled with dyes of different color. Next cells were mixed in proportion 1:1 and 10^6^ of cells were intravenously injected in healthy mice. One hour after injection blood, spleen and lymph nodes were taken to evaluate the proportion of WT and DOK-1/2 DKO CD8^+^ T in each organ. No difference in cell proportion was detected (Fig. S5 A–D). To evaluate conjugate formation, we stained target cells with Cell Trace Violet and effector cells with fluorochrome-coupled CD8 mAb. A co-culture experiment was performed, and conjugate formation was followed during time course (Fig. S6A–C). WT and *Dok1/Dok2* DKO primed CD8^+^ T cells showed the same effector-target conjugate formation.

## Discussion

In this study, we examined the role of DOK1 and DOK2 in naïve and primed CD8^+^ T cells. Primed but not naïve *Dok1*/*Dok2* DKO CD8^+^ T cells had an improved TCR signaling and showed more effector memory subtypes compared to WT CD8^+^ T cells. Building on these results, we hypothesized that DOK1 and DOK2 invalidation in CD8^+^ T cells may be a promising approach to improve their anti-tumor functions and thus subsequent immunotherapy. However, the phenotypic and signaling differences did not translate into a difference in cytotoxic response in vitro (Fig. 3) and did not improve survival of tumor-bearing mice (Fig. S4).

In the present study, we showed that DOK1 and DOK2 regulate subtly in vitro memory CD8^+^ T cell formation. We found that at the end of 5 days of CD8^+^ T cell expansion, we have more effector memory phenotype in *Dok1*/*Dok2* DKO CD8^+^ T cells. Here, we used IL-2 to expand primed CD8 + T cells. By using IL-15, a more effective cytokine to induce a memory phenotype, similar results were reported^30^. We believe that this is due to a slower re-expression of CD62L between day 3 and day 5 when TCR stimulation was canceled. We think that during this period, cells are only proliferating due to IL-2 presence, but T cells subset are starting to “rest” from TCR previous stimulation. At least in CD8^+^ T cells, DOK1 and DOK2 seem to exert their inhibiting role to favorize the activated cells to go back to a “resting” state, but we could not detect any difference at naïve state, not only in signaling but also in phenotypic experiments.

L-selectin (CD62L) controls T-cell migration and is negatively controlled by PI3K-Akt pathway activation^38^. Previously, it was shown that DOK1 negatively controls SDF-1α induced cell migration^39^. Thus, we performed in vivo migration experiments using primed CD8^+^ T cells but we could not find the difference between WT and *Dok1*/*Dok2* DKO T cells.

Previous studies showed major improvements of TCR signaling, proliferation and cytokine production in naïve and memory *Dok1*/*Dok2* DKO CD4^+^ T cells^28,40^. Only few studies were performed on *Dok1*/*Dok2* invalidation in CD8^+^ T cells^30,41^. In agreement with our results WT and *Dok1*/*Dok2* DKO naïve CD8^+^ T cells showed similar signaling upon TCR stimulation. Therefore, we confirm the difference of DOK1 and DOK2 regulation of TCR signaling in CD4^+^ and CD8^+^ naïve T cells. Considering fundamental differences in the role of CD4^+^ and CD8^+^ T cells and their complex functional interplay, it is rational to suggest some differences in TCR signalosome of these cells^42–44^. For example, recently a crucial difference in TCR initiation signaling was revealed showing that LCK binding is stronger to CD8 compared to CD4 coreceptor, leading to more potent intracellular signaling^45^. By using agonist CD3 mAb, we found that primed *Dok1*/*Dok2* DKO CD8^+^ T cells showed an improved TCR signaling by upregulation of pAkt and pErk upon TCR engagement via two kind of experimental approaches. Similar induction of pAkt and pErk are detected by immunoblots at early time points (Fig. 2B & Fig. S2B,C) by using a peptide stimulation, however we were unable to detect a boost of TCR signaling due to the absence of DOK proteins.

In primary T cells, TCR engagement with a CD3 antibody is orders of magnitude stronger than cognate recognition of peptide-MHC^46^. In a T cell line, it has been suggested that after a strong TCR stimulation, possible mechanisms of negative feedback regulation could dampen TCR signaling^47^. This could explain why the upregulation effect of DOK deficiency is only visible upon antibody stimulation. We can notice that a slight pErk upregulation can be detected by phosphoflow analysis (Fig. 2D) upon peptide stimulation in DKO condition. This could be due to the sensitivity experimental method as for flow cytometry, the phosphorylation events are detected at the single cell level^48^.

Naïve and memory T cells have considerable differences in their function physiology and TCR signalosome. Particularly, it was demonstrated that memory CD8^+^ T cells have more CD8-bound Lck than naïve cells and CD4^+^ T cells have less Zap70 and Slp76 phosphorylation upon TCR stimulation, suggesting a faster and more efficient signal transduction pathway in memory T cells^17,19^. These data confirm our findings and suggest some functional or structural differences in CD8^+^ naïve versus primed signalosomes. Further investigation notably using high throughput technologies such as mass spectrometry is needed to decipher these phenomenona^11^.

Compensation mechanisms and signaling re-wiring may also occur in TCR signaling pathway, like the inhibitory TCR signaling protein Csk, normally associated with PAG, could associate with another protein PTPN22 in absence of PAG compensating TCR signaling^40^. DOK1 and DOK2 could be seen as a platform to recruit other inhibitory proteins (RasGAP, SHIP, Csk). Maybe other proteins could compensate the lack of DOK1 and DOK2 when these proteins are totally absent. Thus, methods to downregulate transiently DOK1 and DOK2 in CD8^+^ T lymphocytes by shRNA or CRISPRi-Cas9 techniques may be interesting to avoid these compensation mechanisms and understand more precisely DOK1 and DOK2 regulation.

In this study, we wanted to assess the capacity of TCR signaling inhibitory proteins DOK1 and DOK2 to improve CD8^+^ T cells immunotherapy. In the development of T cell-based immunotherapy the problem of T cell functionality blunting in the tumor microenvironment is crucial. The concept of improving the strength of TCR signaling upon TCR activation is very important to overcome this problem. Therefore, we tested TCR signal inhibiting proteins DOK1 and DOK2 as potential candidates to increase TCR signaling in CD8^+^ T cells. The fact that we found the increase of pErk and pAkt in primed but not naïve CD8^+^ T cells is even advantageous in this context, as only primed CD8^+^ T cells are used for adoptive cell transfer immunotherapies nowadays. Previously, it was shown that inhibition of Akt pathway by rapamycin could improve the generation of memory cells in terms of their quantity and quality^49^. The acquisition of effector functions of CD8^+^ T cells associated with intense Akt signaling impairs the in vivo antitumor efficacy of adoptively transferred cells^50^. The emerging consensus on this question is that central memory tumor-reactive CD8^+^ T cells have an improved antitumor capacity in comparison with effector memory cells^51,52^. Therefore, the activation both pErk and pAkt as we can see in the context of *Dok1*/*Dok2* invalidation would be not advantageous for antitumor capacity of primed CD8^+^ T cells as the positive influence of pErk upregulation would be compensated by the negative influence of effector memory phenotype due to increased pAkt. Probably in the concept of CD8^+^ T cells immunotherapy improvement by acting through TCR signaling inhibiting such polyvalent inhibiting proteins as DOK1 and DOK2 would be excessive, and proteins acting on inhibiting of one specific signaling pathway would fit more. As successful examples of targeting intracellular inhibiting proteins in the context of cancer immunotherapy, we can mention recently adapted for clinical trials CISH, targeting PLC-γ1 and HPK-1, targeting SLP-76^9,22,53^. Both could be associated to Erk pathway improvement, without direct effect on Akt pathway. The role of DOK1 and DOK2 has been reported in another type of cytotoxic lymphocytes, the Natural Killer (NK) cells^54^. It would be interesting to challenge the role of these DOK adaptors in NK cells to eliminate cancer cells.

In summary, our data provided evidence that DOK1 and DOK2 interfere in primed CD8^+^ T cell TCR signaling negative regulation and have impact on memory CD8^+^ T cell formation. We underlined an interesting phenomenon that DOK1 and DOK2 could play a different role in naïve and memory TCR signaling, however based on our model the DOK1/DOK2 adaptor proteins do not appear to be good candidates for CD8^+^ T cell manipulation in immuno-oncology. Therefore, due to complexity of TCR signaling there is a real need of screening studies of invalidation of TCR signaling inhibitory proteins to improve existing CD8^+^ T cell-based immunotherapies.

### Supplementary Information

Supplementary Figures.

## Data Availability

The datasets used and/or analysed during the current study available from the corresponding author on reasonable request.

## References

[1] Arens R, Schoenberger SP (2010). Plasticity in programming of effector and memory CD8 T-cell formation. Immunol. Rev..

[2] Raskov H, Orhan A, Christensen JP, Gogenur I (2021). Cytotoxic CD8(+) T cells in cancer and cancer immunotherapy. Br. J. Cancer.

[3] Waldman AD, Fritz JM, Lenardo MJ (2020). A guide to cancer immunotherapy: From T cell basic science to clinical practice. Nat. Rev. Immunol..

[4] Gajewski TF, Schreiber H, Fu YX (2013). Innate and adaptive immune cells in the tumor microenvironment. Nat. Immunol..

[5] Janicki CN, Jenkinson SR, Williams NA, Morgan DJ (2008). Loss of CTL function among high-avidity tumor-specific CD8+ T cells following tumor infiltration. Cancer Res..

[6] Rabinovich GA, Gabrilovich D, Sotomayor EM (2007). Immunosuppressive strategies that are mediated by tumor cells. Annu. Rev. Immunol..

[7] Vazquez-Cintron EJ, Monu NR, Frey AB (2010). Tumor-induced disruption of proximal TCR-mediated signal transduction in tumor-infiltrating CD8+ lymphocytes inactivates antitumor effector phase. J. Immunol..

[8] Whiteside TL (2006). Immune suppression in cancer: Effects on immune cells, mechanisms and future therapeutic intervention. Semin. Cancer Biol..

[9] Laletin V, Bernard PL, Costa da Silva C, Guittard G, Nunes JA (2023). Negative intracellular regulators of T-cell receptor (TCR) signaling as potential antitumor immunotherapy targets. J. Immunother. Cancer.

[10] Sitaram P, Uyemura B, Malarkannan S, Riese MJ (2019). Beyond the cell surface: Targeting intracellular negative regulators to enhance T cell anti-tumor activity. Int. J. Mol. Sci..

[11] Voisinne G (2019). Quantitative interactomics in primary T cells unveils TCR signal diversification extent and dynamics. Nat. Immunol..

[12] Acuto O, Di Bartolo V, Michel F (2008). Tailoring T-cell receptor signals by proximal negative feedback mechanisms. Nat. Rev. Immunol..

[13] Smith-Garvin JE, Koretzky GA, Jordan MS (2009). T cell activation. Annu. Rev. Immunol..

[14] Boyman O, Letourneau S, Krieg C, Sprent J (2009). Homeostatic proliferation and survival of naive and memory T cells. Eur. J. Immunol..

[15] Kumar BV, Connors TJ, Farber DL (2018). Human T cell development, localization, and function throughout life. Immunity.

[16] Abu-Shah E (2020). Human CD8(+) T cells exhibit a shared antigen threshold for different effector responses. J. Immunol..

[17] Bachmann MF (1999). Developmental regulation of Lck targeting to the CD8 coreceptor controls signaling in naive and memory T cells. J. Exp. Med..

[18] Farber DL, Acuto O, Bottomly K (1997). Differential T cell receptor-mediated signaling in naive and memory CD4 T cells. Eur. J. Immunol..

[19] Hussain SF, Anderson CF, Farber DL (2002). Differential SLP-76 expression and TCR-mediated signaling in effector and memory CD4 T cells. J. Immunol..

[20] Kannan A, Huang W, Huang F, August A (2012). Signal transduction via the T cell antigen receptor in naive and effector/memory T cells. Int. J. Biochem. Cell Biol..

[21] Guittard G (2018). The Cish SH2 domain is essential for PLC-gamma1 regulation in TCR stimulated CD8(+) T cells. Sci. Rep..

[22] Palmer DC (2015). Cish actively silences TCR signaling in CD8+ T cells to maintain tumor tolerance. J. Exp. Med..

[23] Carpino N (1997). p62(dok): A constitutively tyrosine-phosphorylated, GAP-associated protein in chronic myelogenous leukemia progenitor cells. Cell.

[24] Favre C (2003). DOK4 and DOK5: New Dok-related genes expressed in human T cells. Genes Immun..

[25] Guittard G (2018). Evolutionary and expression analyses reveal a pattern of ancient duplications and functional specializations in the diversification of the Downstream of Kinase (DOK) genes. Dev. Comp. Immunol..

[26] Mashima R, Hishida Y, Tezuka T, Yamanashi Y (2009). The roles of Dok family adapters in immunoreceptor signaling. Immunol. Rev..

[27] Yamanashi Y, Baltimore D (1997). Identification of the Abl- and rasGAP-associated 62 kDa protein as a docking protein, Dok. Cell.

[28] Yasuda T (2007). Dok-1 and Dok-2 are negative regulators of T cell receptor signaling. Int. Immunol..

[29] Dong S (2006). T cell receptor for antigen induces linker for activation of T cell-dependent activation of a negative signaling complex involving Dok-2, SHIP-1, and Grb-2. J. Exp. Med..

[30] Laroche-Lefebvre C (2016). Dok-1 and Dok-2 regulate the formation of memory CD8+ T cells. J. Immunol..

[31] van Dijk TB (2000). Stem cell factor induces phosphatidylinositol 3′ -kinase-dependent Lyn/Tec/Dok-1 complex formation in hematopoietic cells. Blood.

[32] Zhao M, Janas JA, Niki M, Pandolfi PP, Van Aelst L (2006). Dok-1 independently attenuates Ras/mitogen-activated protein kinase and Src/c-myc pathways to inhibit platelet-derived growth factor-induced mitogenesis. Mol. Cell. Biol..

[33] Yasuda T (2004). Role of Dok-1 and Dok-2 in myeloid homeostasis and suppression of leukemia. J. Exp. Med..

[34] Overwijk WW (2003). Tumor regression and autoimmunity after reversal of a functionally tolerant state of self-reactive CD8+ T cells. J. Exp. Med..

[35] Eil R (2016). Ionic immune suppression within the tumour microenvironment limits T cell effector function. Nature.

[36] Dong Z (2012). The adaptor SAP controls NK cell activation by regulating the enzymes Vav-1 and SHIP-1 and by enhancing conjugates with target cells. Immunity.

[37] Guittard G (2015). Absence of both Sos-1 and Sos-2 in peripheral CD4(+) T cells leads to PI3K pathway activation and defects in migration. Eur. J. Immunol..

[38] Sinclair LV (2008). Phosphatidylinositol-3-OH kinase and nutrient-sensing mTOR pathways control T lymphocyte trafficking. Nat. Immunol..

[39] Okabe S (2005). Stromal cell-derived factor-1alpha/CXCL12-induced chemotaxis of T cells involves activation of the RasGAP-associated docking protein p62Dok-1. Blood.

[40] Davidson D (2016). The Csk-associated adaptor PAG inhibits effector T cell activation in cooperation with phosphatase PTPN22 and Dok adaptors. Cell Rep..

[41] Lahmidi S, Yousefi M, Dridi S, Duplay P, Pearson A (2017). Dok-1 and Dok-2 are required to maintain herpes simplex virus 1-specific CD8(+) T cells in a murine model of ocular infection. J. Virol..

[42] Castellino F, Germain RN (2006). Cooperation between CD4+ and CD8+ T cells: When, where, and how. Annu. Rev. Immunol..

[43] Laidlaw BJ, Craft JE, Kaech SM (2016). The multifaceted role of CD4(+) T cells in CD8(+) T cell memory. Nat. Rev. Immunol..

[44] Seder RA, Ahmed R (2003). Similarities and differences in CD4+ and CD8+ effector and memory T cell generation. Nat. Immunol..

[45] Horkova V (2020). Dynamics of the coreceptor-LCK interactions during T cell development shape the self-reactivity of peripheral CD4 and CD8 T cells. Cell Rep..

[46] Germain RN, Stefanova I (1999). The dynamics of T cell receptor signaling: Complex orchestration and the key roles of tempo and cooperation. Annu. Rev. Immunol..

[47] Chua XY, Salomon A (2021). Ovalbumin antigen-specific activation of human T cell receptor closely resembles soluble antibody stimulation as revealed by BOOST phosphotyrosine proteomics. J. Proteome Res..

[48] Firaguay G, Nunes JA (2009). Analysis of signaling events by dynamic phosphoflow cytometry. Sci. Signal.

[49] Araki K (2009). mTOR regulates memory CD8 T-cell differentiation. Nature.

[50] Gattinoni L (2005). Acquisition of full effector function in vitro paradoxically impairs the in vivo antitumor efficacy of adoptively transferred CD8+ T cells. J. Clin. Invest..

[51] Klebanoff CA (2017). Inhibition of AKT signaling uncouples T cell differentiation from expansion for receptor-engineered adoptive immunotherapy. JCI Insight.

[52] Klebanoff CA (2005). Central memory self/tumor-reactive CD8+ T cells confer superior antitumor immunity compared with effector memory T cells. Proc. Natl. Acad. Sci. U. S. A..

[53] Liu J (2019). Critical role of kinase activity of hematopoietic progenitor kinase 1 in anti-tumor immune surveillance. PLoS One.

[54] Celis-Gutierrez J (2014). Dok1 and Dok2 proteins regulate natural killer cell development and function. EMBO J..

