# Supplementary material for: DOK1 and DOK2 regulate CD8 T cell signaling and memory formation without affecting tumor cell killing
Source: Sci Rep. 2024 Jul 1;14:15053. doi: 10.1038/s41598-024-66075-0 (PMC11220026; doi:10.1038/s41598-024-66075-0)
Supplement: Supplementary file 1 — Supplementary Figures. [file 41598_2024_66075_MOESM1_ESM.docx]

**Supplementary Figures / Information for**

Vladimir Laletin^1^, Pierre-Louis Bernard^1^, Camille Montersino^2^, Yuji Yamanashi ^3^, Daniel Olive^1^, Rémy Castellano^2^, Geoffrey Guittard^1^* and Jacques A. Nunès^1^*^,🖂^

* These authors contributed equally

^🖂^ Corresponding author: [jacques.nunes@inserm.fr](mailto:jacques.nunes@inserm.fr)

**^1^** Centre de Recherche en Cancérologie de Marseille, CRCM, Immunity and Cancer Team, Institut Paoli-Calmettes, Inserm, CNRS, Aix Marseille Univ, Marseille, France.

**^2^** Centre de Recherche en Cancérologie de Marseille, CRCM, TrGET pre-clinical assay platform, Institut Paoli-Calmettes, Inserm, CNRS, Aix Marseille Univ, Marseille, France.

**^3^** Division of Genetics, The Institute of Medical Science, The University of Tokyo, 4-6-1 Shirokanedai, Minato-ku, Tokyo, 108-8639, Japan.


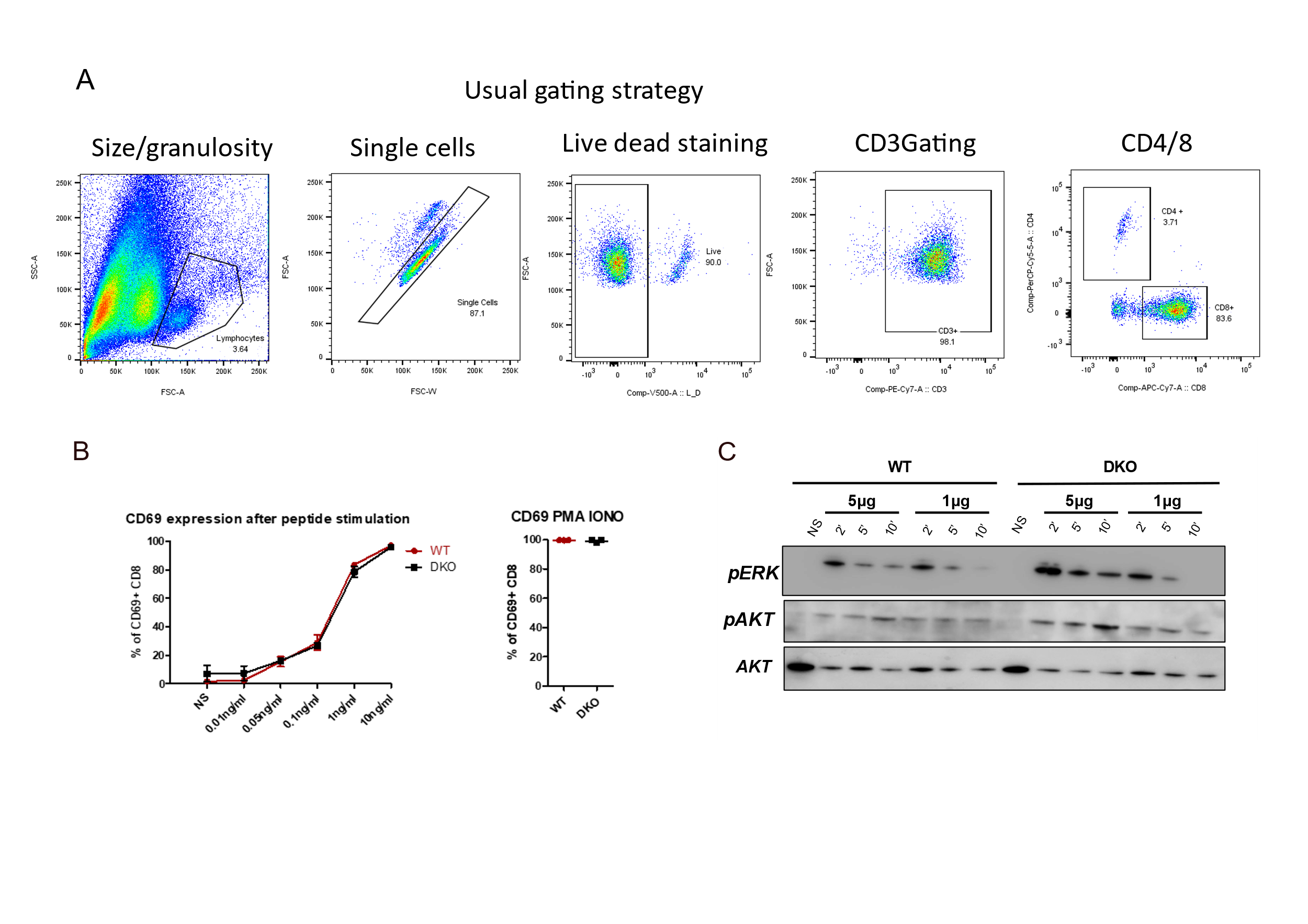


**Supplementary Figure 1. CD8 gating strategy by flow cytometry. CD69 expression upon peptide stimulation and validation by immunoblotting of TCR signaling in primed CD8+ T cells.**

A) Gating strategy to study CD4+ and/or CD8+ T cells. B) CD69 expression after 3hrs indicated concentration peptide stimulation of primed CD8+ T cells. C) Primed CD8+ T cells incubated for 20 min at 4°C with biotinylated CD3ε mAb at 5µg/ml and 1µg/ml. Then, cells were washed and stimulated for the indicated time by adding streptavidin at 37°C. Full-length blots are shown at the end of this file. Representative immunoblot of 3 experiments is shown.


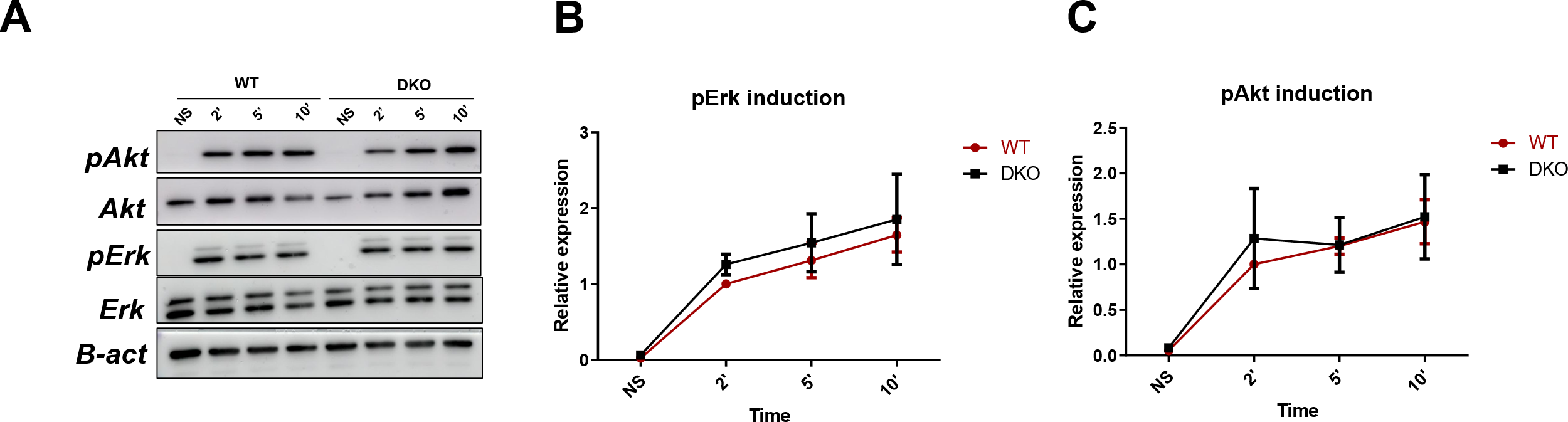


**D**

**E**

**D**


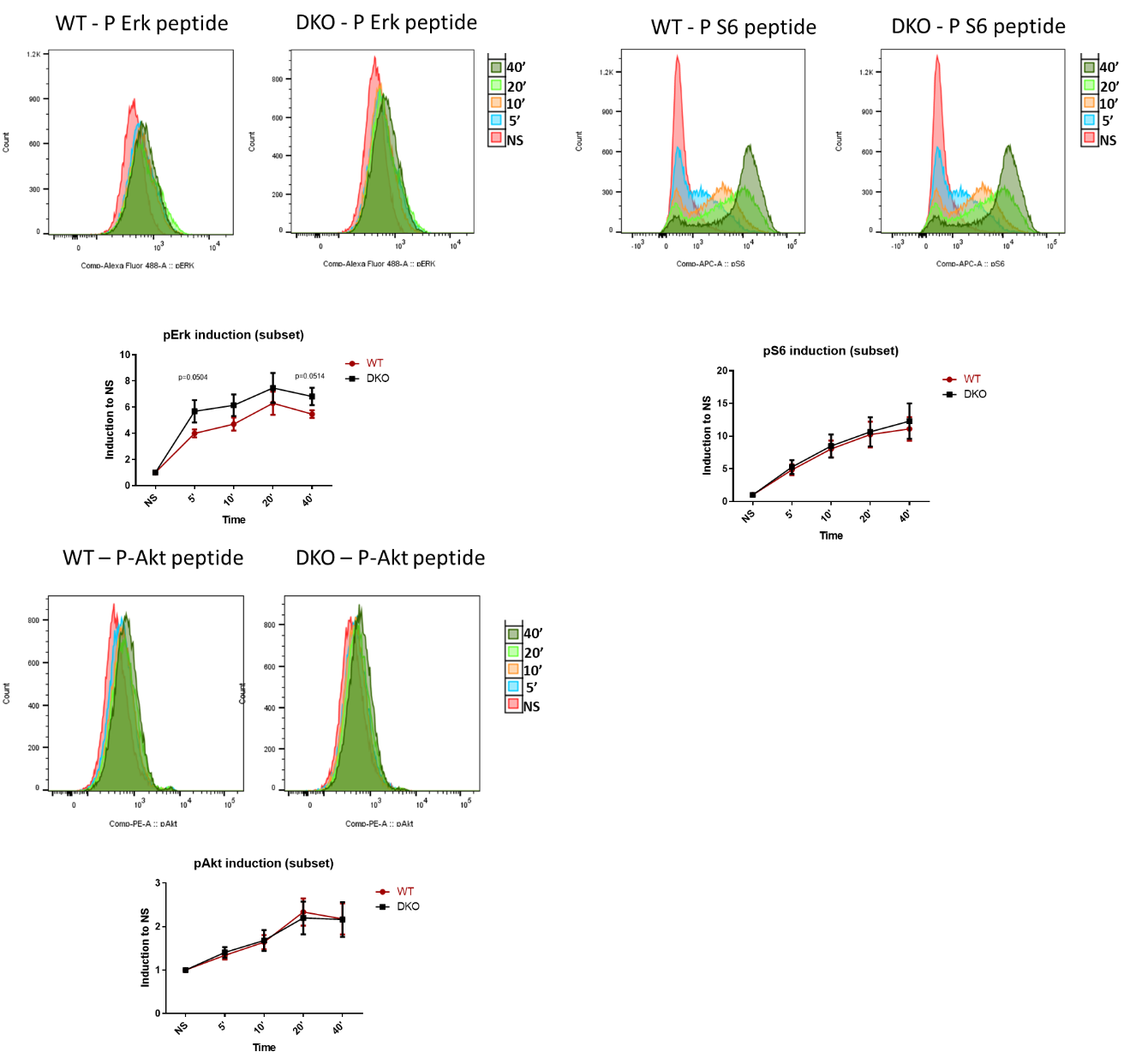


**F**

**Supplementary Figure 2. Upon peptide stimulation, only a slight increase of ERK phosphorylation is detected by phosphoflow analysis in *Dok1/Dok2* DKO compared to WT primed CD8+ T cells.** A) Primed CD8+ T cells were stimulated by hgp-100 peptide 1000ng/ml. Representative immunoblot of primed cells stimulated with hgp-100 for 2, 5 and 10 min. Normalized quantification of pErk (B) and pAkt (C) induction is shown by the entire proteins (respectively Erk-1/2 (Erk) and Akt) as the control loading (n=4). D, E, F) Representative plots of pErk, pAkt and pS6 phosphoflow experiments using peptide stimulation were shown.


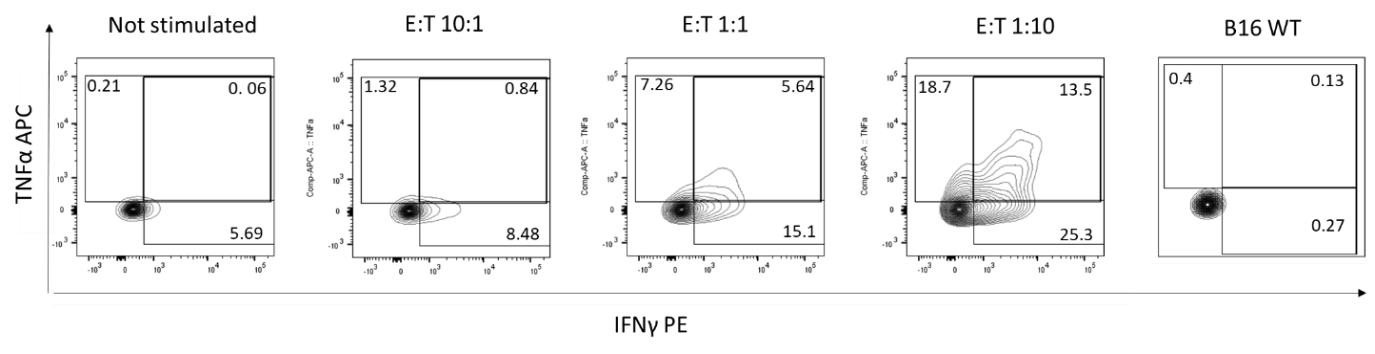


**Supplementary Figure 3. Validation of cytotoxicity against the B16^hgp100^ cell line.**

Contour plots showing cytokine production of unstimulated primed WT CD8+ T cells after 4h of stimulation with B16-WT or B16 expressing hgp100 in different E:T ratios.


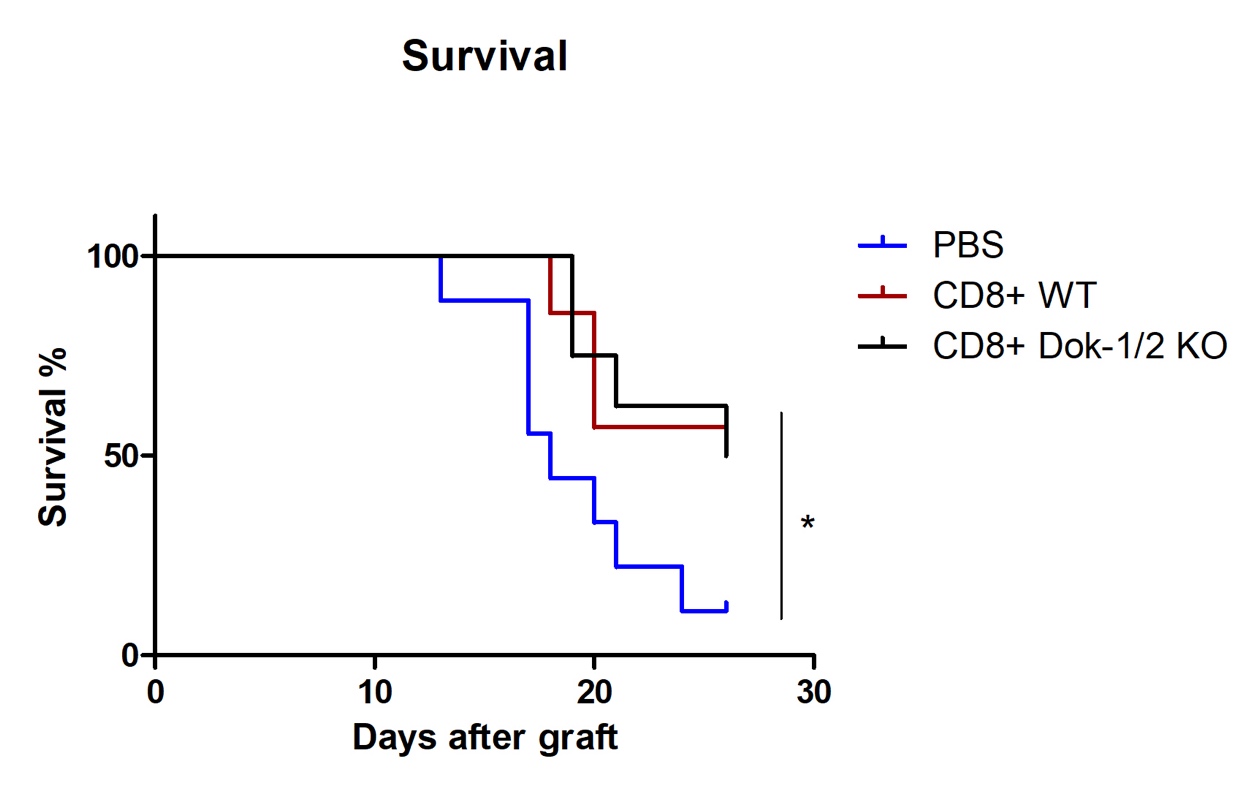


**Supplementary Figure 4. *Dok1/Dok2* DKO adoptive primed CD8+ T cell transfer do not improve survival in cancer model.**

Kaplan-Meier curve of mouse survival after B16 hgp100 implantation and treatment with PBS, WT or *DOK*/*DOK2* DKO primed CD8+ T cells (n = 9 for “PBS” condition and n = 8 for the two adoptive cell transfer conditions). IL-2 was injected at D10, D11, D12 in the three different conditions. Mantel Log-rank test was used to compare survival curves between PBS and WT or DKO adoptive cell transfer (*, p<0,05).


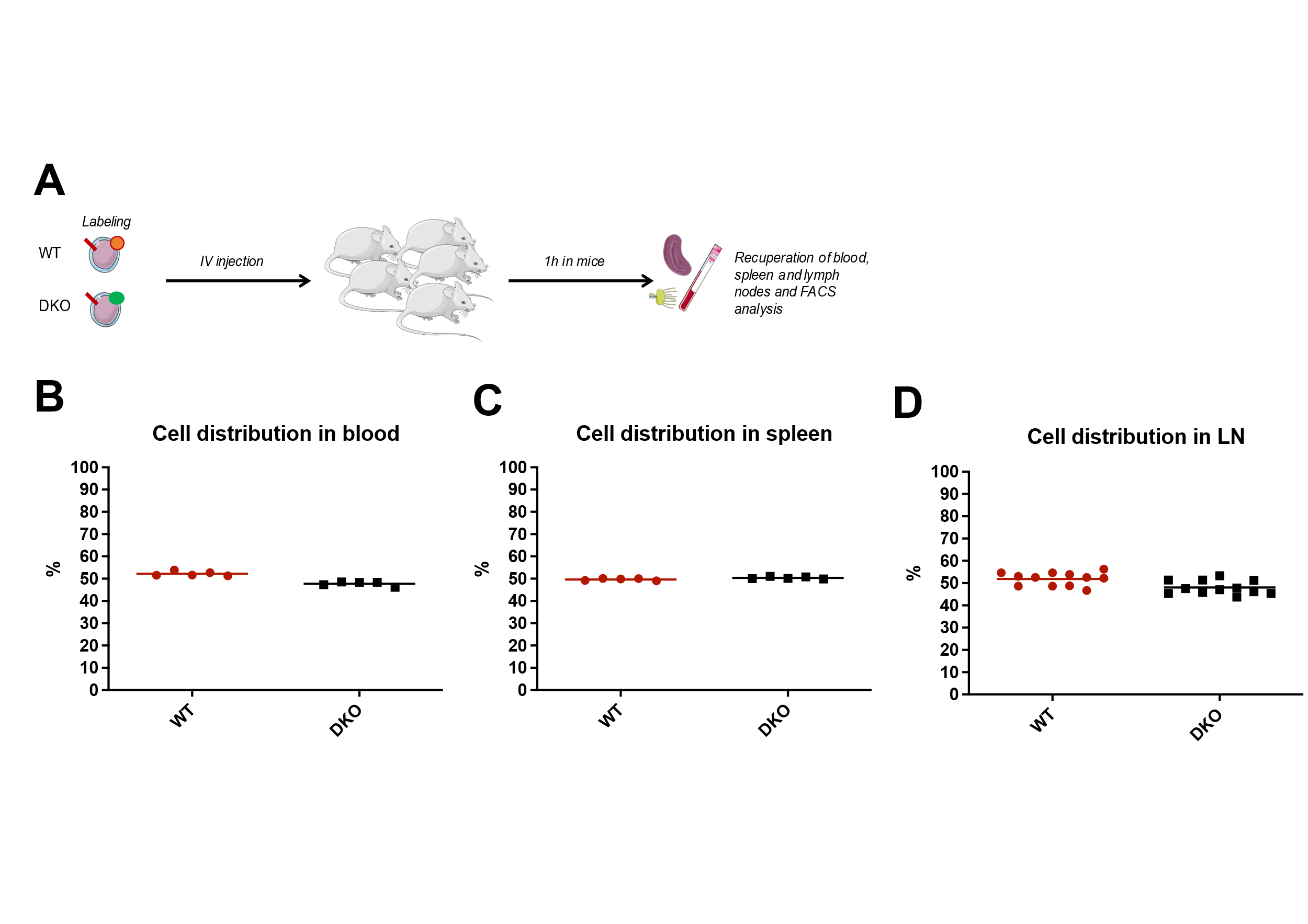


**Supplementary Figure 5. *Dok1*/*Dok2* DKO does not affect primed CD8^+^ T cells *in vivo* migration.**

A) Experimental protocol of *in vivo* primed CD8^+^ T cell migration. WT and DKO cell distribution in blood (B), spleen (C) and lymph nodes (D) is shown (n=5).


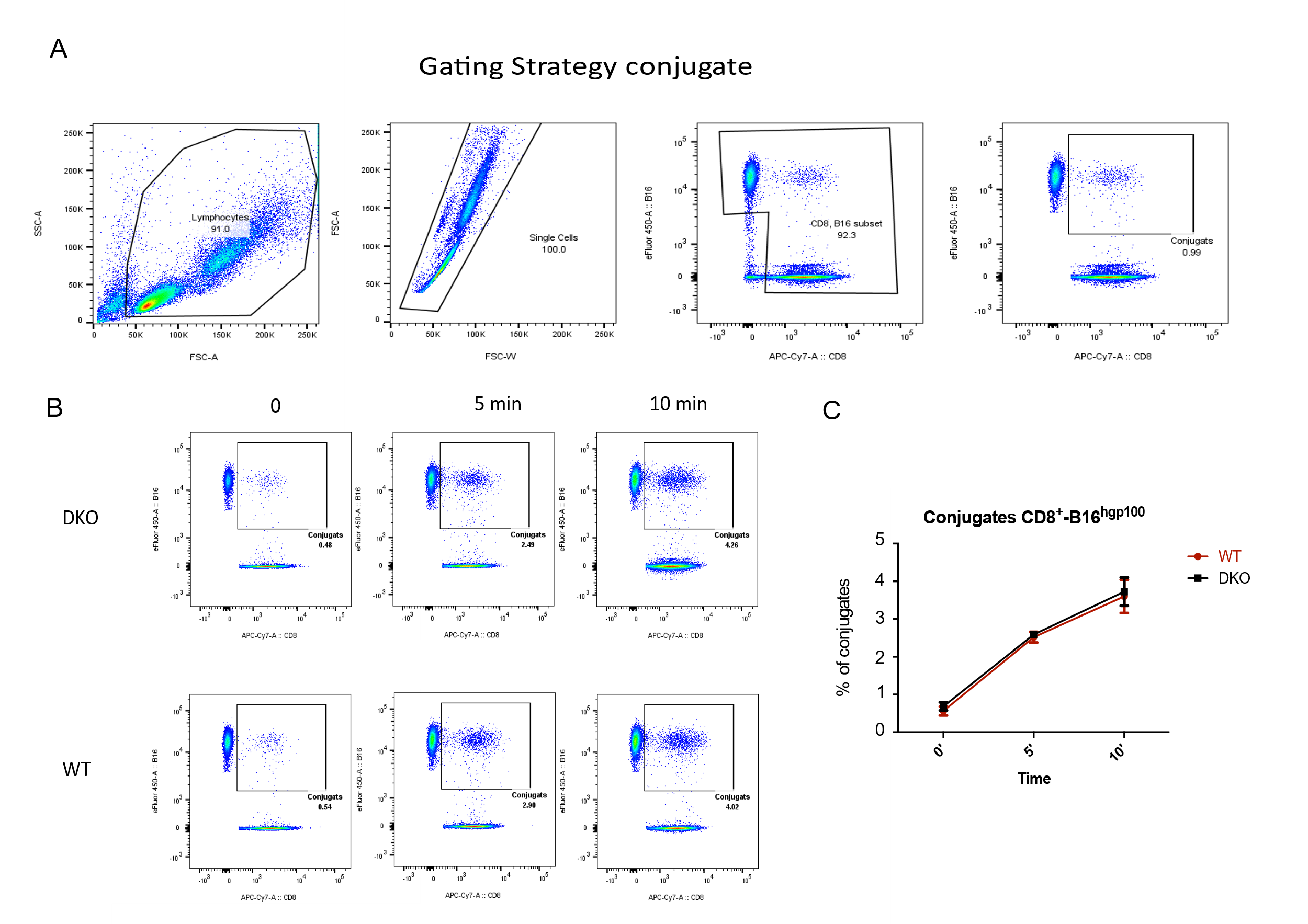


**Supplementary Figure 6. *Dok1*/*Dok2* DKO does not affect CD8^+^ T cells conjugate formation with tumor cells.**

Conjugate formation between primed CD8^+^ T cells and B16 hgp100 cells at 1:1 ratio after contact and co-culture for 0, 5 and 10 min measured by flow cytometry. A) Gating strategy to detect cell conjugates. B) Representative scatter plots of WT and DKO conjugates at indicated times. C) Conjugate formation between primed CD8^+^ T cells and B16 hgp100 cells at 1:1 ratio after contact and co-culture for 5 and 10 min measured by flow cytometry (n=4).

**Full-length gels and blots (Figures 2A-B, S1, S2A)**


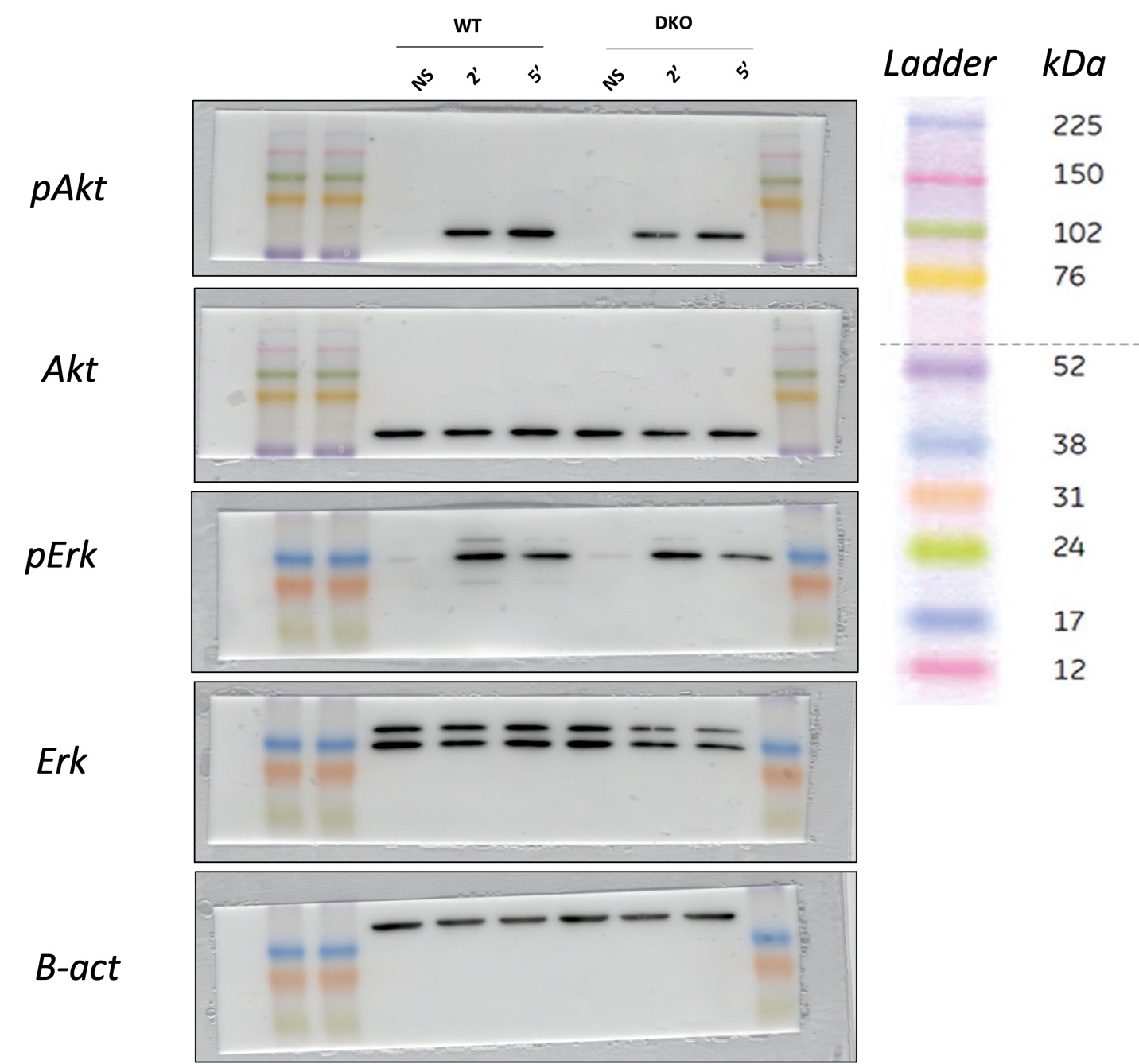


Full-length blots for Figure 2A


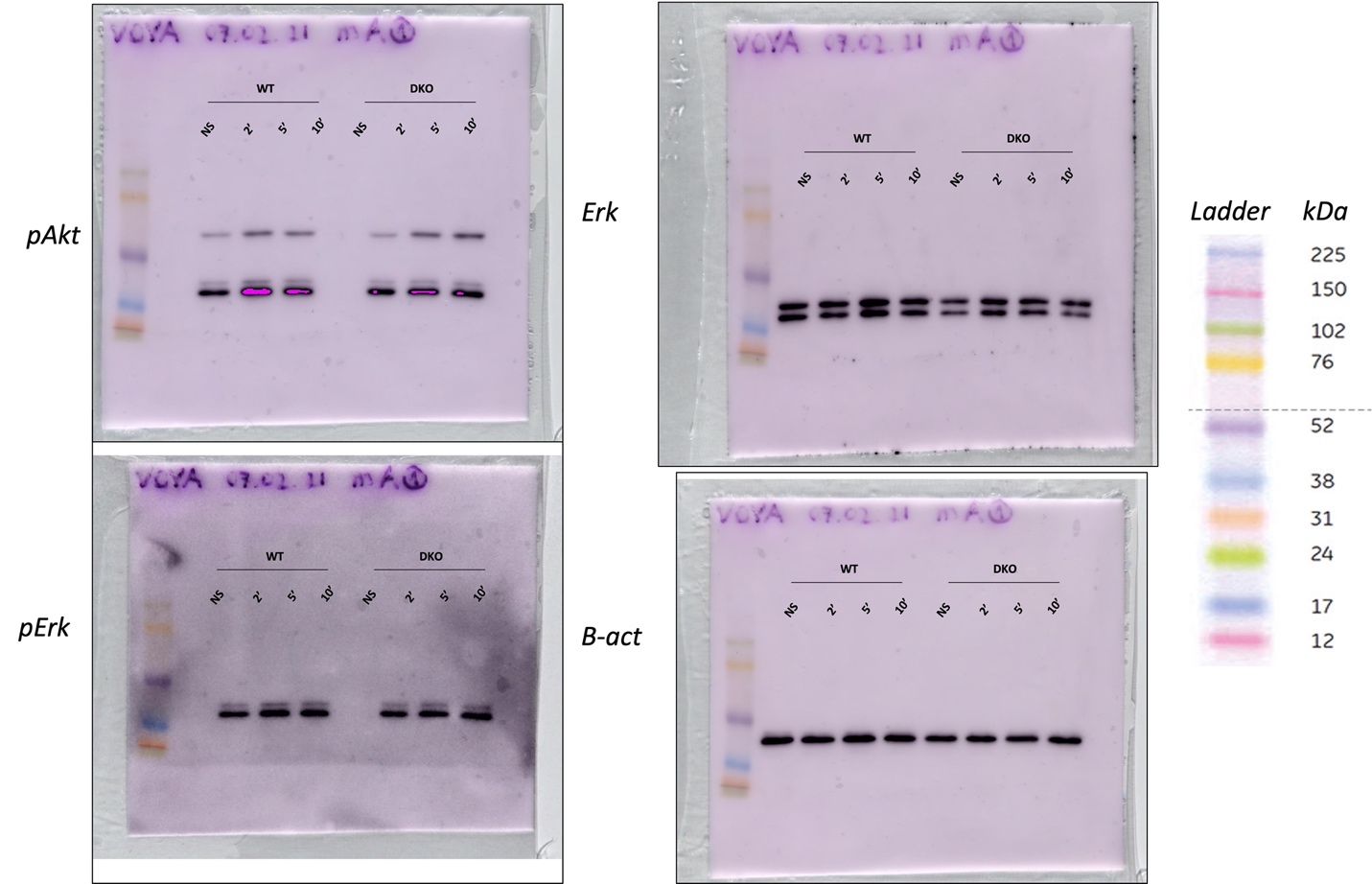


Full-length blots for Figure 2B


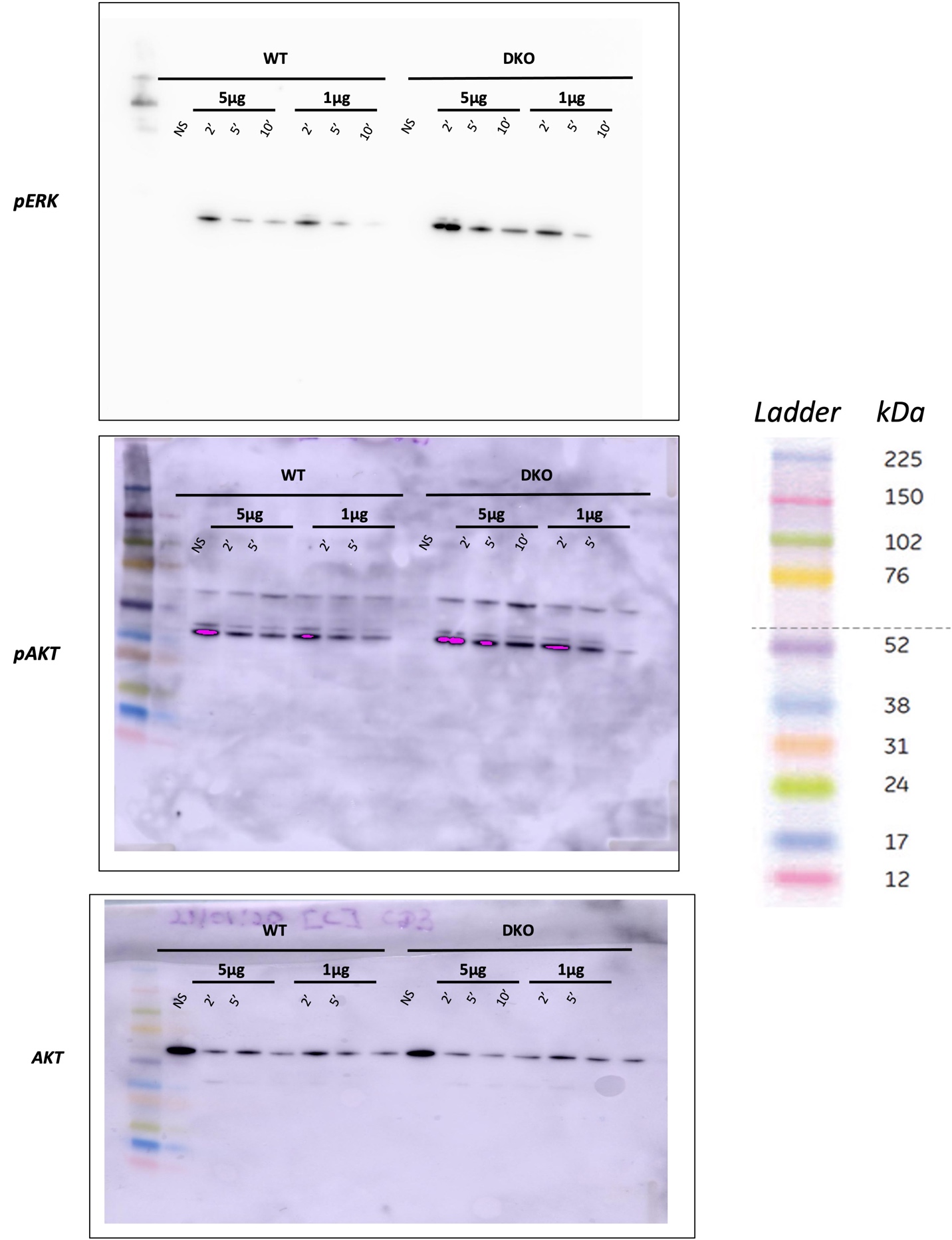


Full-length blots for Supplementary Figure 1C

- for pAKT immunoblot, pAKT is corresponding to the upper band around 60 kDa

- for pAKT and AKT immunoblots, the wells without a time label are corresponding to 10 min


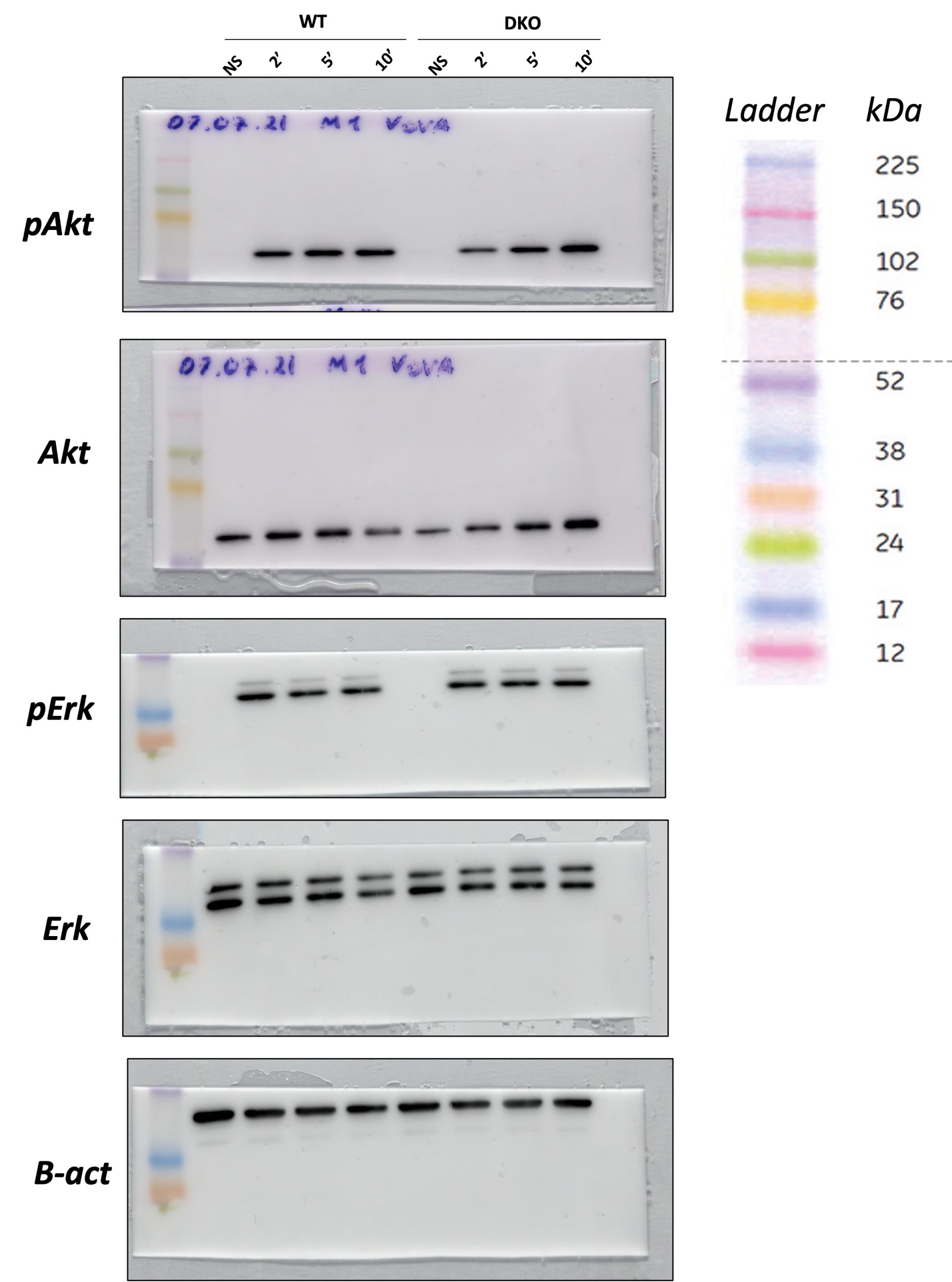


Full-length blots for Supplementary Figure 2A
